# Distribution of critically ill patients (CIP) with severe sepsis (SS) according to the major diagnostic categories (MDC) of the diagnosis-related groups (DRG)

**DOI:** 10.1186/2197-425X-3-S1-A735

**Published:** 2015-10-01

**Authors:** J Ruiz Moreno, E González Marín, MJ Esteve Paños, M Juliá Amill, N Suárez Álvarez, M Moral Guiteras, F Baigorri González, A Artigas Raventós

**Affiliations:** QuirónSalud Hospital Universitario Sagrat Cor, Critical Care Department, Barcelona, Spain; Hospital de Sabadell & QuirónSalud Hospital Universitario Sagrat Cor, Critical Care Department, Sabadell, Spain

## Introduction

Apart from financial purposes (cost accounting), DRG as case - mix system are not used for obtaining competitive advantage. MDC can be useful to classify the SS; if so, it is possible to assign each focus of sepsis to a specific category, which is important from a socio-economic perspective.

## Objectives

- Identify which MDCs encompasses a greater or lesser number of CIPs con SS.
- Find out which MDC incorporating CIPs with SS carries a higher an average relative weight (RW).

## Methods

- Type of Study: prospective, analytical, longitudinal, and observational
- Period: January 1-2011 / June 30-2014 (42 months)

## SETTING

Medical/Surgical ICU

- Population: 2559 CIPs admitted consecutively to the ICU; sample: 484 CIPs.
- Exclusión criteria: CIPs < 16 y., major burn CIPs, incomplete clinical documentation, and voluntary discharge.
- DRG AP-DRG 25.0 version (684 DRG are grouped into 25 MDC and 1 extra MDC).
- Depending on the focus of sepsis, SS related to MDC '0' (precategoría) are transferred to another MDC.
- MDC: 1 (neurology), 2 (eye), 3 (ear, nose, mouth, throat), 4 (respiratory), 5 (circulatory), 6 (digestive, 7 (hepatobiliary & pancreas), 8 (musculoskeletal & connective), 9 (skin & breast), 10 (endocrine), 11 (urinary tract), 12 (male reproductive), 13 (female reproductive), 14 (pregnancy & childbirth), 15 (newborn), 16: (blood & immunological), 17 (mMyeloproliferative), 18 (infectious), 19 (mental), 20 (alcohol / drug), 21 (Injuries & poison), 22 (burns), 23 (factors influencing health status), 24 (HIV), 25 (PLT), 0 (PreMed, miscellany)

## Results

See Tables 1, 2 and 3.

**Table 1:** Results I

	Global	%	SS	%	no SS	%	p value
CIPs	2559	100	484	18,8	2075	81,1	
Age	65,9		73,5		64,1		0,001
Mortality	159	6,21	119	24,6	63	3,0	0,001
RW	4,21		7,9		3,35		0,001

**Table 2:** Results II.

MDC	SS	%	average RW
1	8	1,7	8,4598
4	114	24,0	11,0677
5	30	6,3	6,1961
6	152	32,0	9,9382
7	94	19,8	4,5068

**Table 3:** Results III.

MDC	SS	%	average RW
8	14	2,9	5,1191
9	8	1,7	4,0320
11	22	4,6	5,1687
18	33	6,9	3,7598
Global	475	100	7,9815

Excluded MDCs (< 8 DRG with SS): '2', '3', '4', '12','13', '14', '15', '16', '17', '19', '20', '21', '22', '23', '24' y '25'

## Conclusions

- 16 MDC do not identify SS (or less than 8 DRG).
- MDC '6', '4' and '7' carry more SS, that, it respectively, correspond to the septic focus abdominal, respiratory, and biliopancreatic.
- MDC '4' and '6' show the highest RW
